# Effectiveness of CO_2_-insufflated endoscopic submucosal dissection with the duodenal balloon occlusion method for early esophageal or gastric cancer: a randomized case control prospective study

**DOI:** 10.1186/1471-230X-12-37

**Published:** 2012-04-24

**Authors:** Hirohito Mori, Hideki Kobara, Shintaro Fujihara, Noriko Nishiyama, Kunihiko Izuishi, Masaomi Ohkubo, Kazi Rafiq, Yasuyuki Suzuki, Tsutomu Masaki

**Affiliations:** 1 Department of Gastroenterology and Neurology, 1750-1 Ikenobe, Miki, Kita, Kagawa, 761-0793, Japan; 2 Department of Gastroenterological Surgery, 1750-1 Ikenobe, Miki, Kita, Kagawa, 761-0793, Japan; 3 Department of Radiology, 1750-1 Ikenobe, Miki, Kita, Kagawa, 761-0793, Japan; 4 Department of Pharmacology, Kagawa Medical University School of Medicine, 1750-1 Ikenobe, Miki, Kita, Kagawa, 761-0793, Japan

## Abstract

**Background:**

Endoscopic submucosal dissection (ESD) has typically been performed using air insufflation. Recently, however, insufflation of CO_2_ has been increasingly used to avoid complications. This prospective study was designed to compare the CO_2_ concentration, intestinal volume, and acid–base balance using the duodenal balloon procedure.

**Methods:**

From June 2010 to February 2011, we enrolled 44 patients with esophageal or gastric cancer and randomly allocated them into two groups. We compared 22 patients undergoing CO_2_-insufflated ESD with a balloon placed into the duodenal bulb (duodenal balloon group) and 22 patients undergoing regular CO_2_-insufflated ESD (regular group). Three-dimensional computed tomography was performed before and after the procedure to measure intestinal volume. CO_2_ concentrations were measured every 10 minutes. The visual analogue system (VAS) scores for postoperative symptoms were recorded, and pH was measured immediately after the procedure. This was a prospective case control study randomized by the sealed envelope method.

**Results:**

Intestinal CO_2_ gas volume before and after ESD was lower in the duodenal balloon group than in the regular group (*P* = 0.00027). The end-tidal CO_2_ level was significantly lower in the duodenal balloon group than in the regular group (*P* = 0.0001). No significant differences in blood ΔpH were found between the two groups. The VAS score for the occurrence of nausea due to abdominal distension after ESD indicated a significant difference (*P* = 0.031).

**Conclusions:**

ESD using the duodenal balloon occlusion method is effective for reduction of post-ESD intestinal CO_2_ gas volume, resulting in a lower total amount of CO_2_ insufflation during ESD and reducing harmful influences on the human body to some extent.

## Background

Endoscopic submucosal dissection (ESD) for gastrointestinal malignancy has been increasingly performed in Japan, and its technical basis is almost completely established [1-4]. The procedure is also covered by the health insurance system in Japan. However, ESD is technically complicated [5,6], requires a high level of endoscopic skill, and has been associated with serious complications [7,8]. Although insufflation of air has traditionally been used in ESD, there are many risks, such as air embolism. As an alternative to air, insufflation of CO_2_ has been used in regular colonoscopy and colorectal ESD which has been proven effective [9-17]. Insufflation of CO_2_ is also available to help maintain a stable respiratory and hemodynamic state during ESD by preventing pneumoperitoneum when perforation occurs [18-21] and reducing post-ESD symptoms, such as nausea due to abdominal distension. In addition, given recent issues related to air insufflation, such as air embolism, insufflation of CO_2_ is expected to become increasingly more common.

The present study was a prospective comparison of changes in CO_2_ concentration during the procedure and changes in intestinal volume and acid–base balance using the duodenal balloon occlusion method.

## Methods

### Patients

We enrolled 44 patients who were diagnosed with early gastric or esophageal cancer and demonstrated expanding indications for ESD by the gastric cancer treatment guidelines of the Japanese Gastric Cancer Association (JGCA). The expanding indications of ESD are as follows: mucosal differentiated adenocarcinoma of any size without ulceration, SM1-invaded differentiated adenocarcinoma within 3 cm in diameter, and mucosal undifferentiated adenocarcinoma within 2 cm in diameter without ulceration. All patients underwent ESD using CO_2_ insufflation.

We randomly allocated 44 patients (34 males and 10 females) who underwent ESD for early esophageal or gastric cancer into two groups by the sealed enveloped method from June 2010 to February 2011 (Table 1). The exclusion criteria were a past history of a duodenal ulcer or the presence of an ulcer scar.

**Table 1:** No significant differences were found with respect to age, gender, location of lesion, diameter of resected lesion, or procedure time

	**Duodenal balloon group ** **n = 21**	**Regular group ** **n = 22**	**P value**
Age(years) (mean ± SD)	50 ~ 79 (69.0 ± 9.8)	40 ~ 85 (71.6 ± 12.7)	NS**
Gender (M/F)	17/4	16/6	NS*
Location of lesion (Eso/U/M/L)	4/8/4/5	2/8/5/7	NS**
Size of resected specimen (mm)	17 ~ 80 (36.1 ± 19.3)	17 ~ 70 (38.8 ± 17.9)	NS**
Procedure time (min)	105 ~ 180 (145.6 ± 26.6)	30 ~ 270 (127.0 ± 67.3)	NS***

*Fisher’s exact t-test,**Unpaired t-test,***Mann–Whitney U-test.

We informed all patients of this study’s aim and method. Full details of patients’ informed consents were followings: using the balloon for esophageal variceal injection sclerotherapy as duodenal occlusion balloon. An overtube was used to allow backflow of CO_2_ gas from the stomach to the oral cavity for evacuation from the mouth, preventing the gas from flowing into the trachea and lungs. Under putting the balloon, we would perform ESD. We informed these procedures and side effects, and obtained consents from all patients in written forms. The study protocol was reviewed and approved by the Kagawa university ethical committee according to the Declaration of Helsinki. All subjects gave written, informed consent before any study related procedures were performed.

### Study design

This was a single-center, randomized, case control study. The objectives were to examine the intestinal CO_2_ gas volume measured by three-dimensional computed tomography (3DCT) before and after ESD using the duodenal balloon occlusion method and determine the effect on certain parameters between in the duodenal balloon group and regular group.

We performed ESD on 44 patients. We performed CO_2_-insufflated ESD with a duodenal balloon in 22 patients (duodenal balloon group) and regular CO_2_-insufflated ESD without the balloon in 22 patients (regular group). The duodenal balloon occlusion method is described in following section. 3DCT was performed the day before ESD and just after the ESD to measure intestinal gas volume. CO_2_ concentrations were measured every 10 minutes by a capnometer (end-tidal CO_2_; PETCO_2_) every 10 min until the end of ESD ( from 0 to 120 min; however, if ESD was finished earlier, measurement was terminated; if ESD lasted longer, measurement was terminated at 120 min. that’s because under the stable CO2 insufflation, ΔPETCO2 doesn’t change so much ). Arterial blood gas pH was measured in both groups just before ESD, and after ESD subsequently to 3DCT (about 15 minutes later after ESD finished). The visual analogue system (VAS) scores for postoperative symptoms were recorded after the procedure.

### Duodenal balloon occlusion method

**Figure 1:**
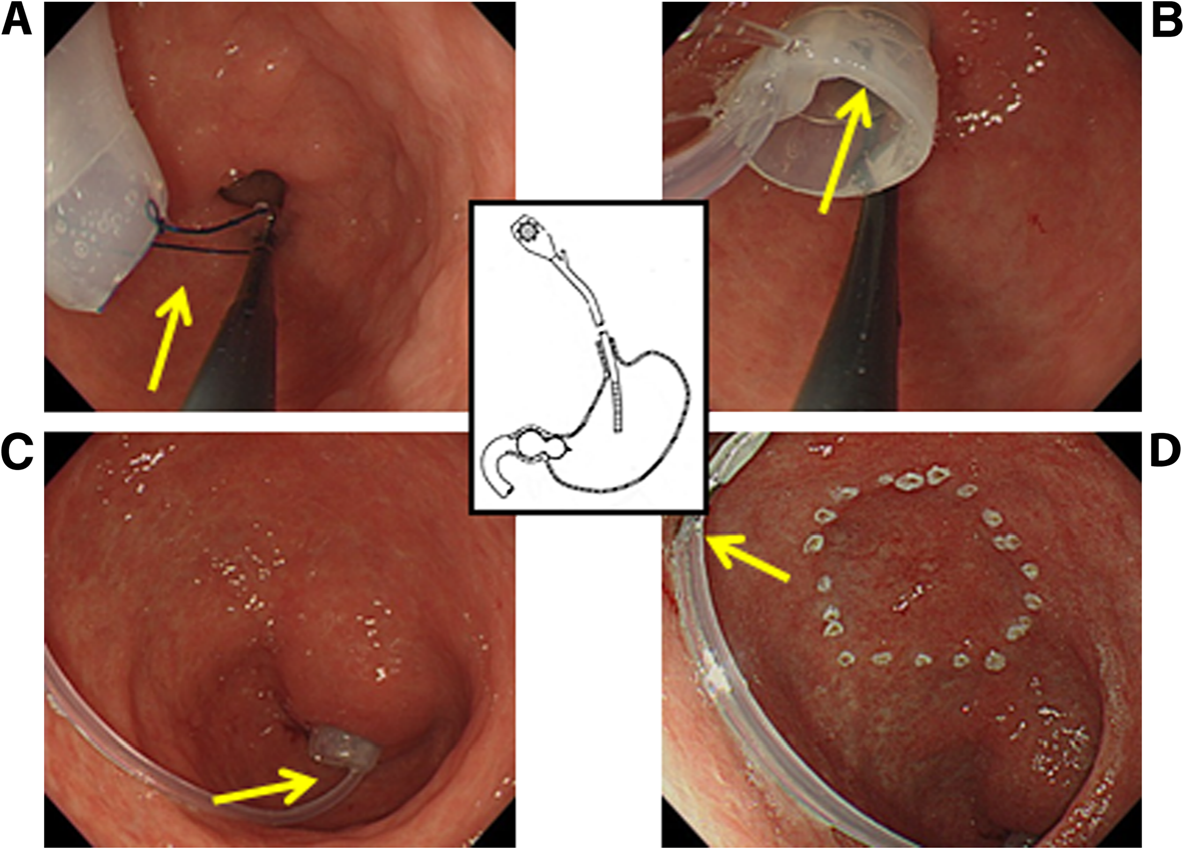
**(A) A circular ring was made with a nylon thread at the tip of the balloon to create a better grip.** (B) This circular ring was grasped with a gripping forceps and inserted into the duodenal bulb. (C) The balloon was dilated with 60 to 70 mL of air. (D) The intragastric insufflation tube was clipped onto the gastric wall to prevent the insufflation tube from interfering with the lesion to be removed.

We used the balloon for esophageal variceal injection sclerotherapy. We made a circular ring with a nylon thread at the tip of the balloon to create a better grip, grasped this circular ring with a gripping forceps, and inserted it through the overtube into the duodenal bulb. We infused an appropriate amount of air as calculated for previous gastric fluoroscopy (60–70 mL on average) to fix the balloon (Figure 1). During the procedure, an overtube was used to allow backflow of CO_2_ gas from the stomach to the oral cavity for evacuation from the mouth, preventing the gas from flowing into the trachea and lungs. This overtube with no valve could evacuate the backflowing gas from the body.

### 3DCT (intestinal gas volume)

Reconstructions of 3DCT and measurements of intestinal gas volume were performed and calculated by radiology technician Dr. Masaomi Ohkubo. 3DCTs were reconstructed from raw CT images by volume rendering (VR) technique. The cut off level of VR was set at -400HU. The voltage was 120 kV and the electric current was 200 mA. Raw Slice thickening was 1 mm and reconstruction was 0.8 mm, and using these parameters,the accuracy of calculated intestinal volumes was 99.5%.

### Outcomes

The primary outcome was the intestinal volume measured by 3DCT before and after ESD using the duodenal balloon occlusion method compared with that of the regular group. The secondary outcome was the intraoperative change in CO_2_ concentration between the two groups as measured by a capnometer (end-tidal CO_2_; PETCO_2_) every 10 min until the end of ESD. Differences in arterial blood gas pH between the two groups were measured immediately before and after ESD. Patients’ abdominal symptoms after ESD as scored using the VAS ranged from 0 (no symptoms) to 10 (severe symptoms).

### Statistical analysis and sample size

The null hypothesis was that the intestinal CO_2_ gas volumes measured by 3DCT before and after ESD using the duodenal balloon occlusion method would be identical to those of the regular group.

A previous study using air insufflation with or without duodenal balloon occlusion has been reported [22]. We referred to the study and calculated the target number of 64 subjects per group based on G*Power using the effective size of 0.5. An effective size of 0.8 resulted in a target number of 25. Based on these numbers, we decided to use a target number of 44 to 45.

### Randomizations and procedures

This was a single-center, randomized, case control study. Patients were randomly allocated by the sealed envelope method. The randomization was achieved using sealed numbered envelopes, prepared previously by Dr. H. K. The randomization code was not broken until the study was completed. All investigators attended a study meeting before the study and received instructions on performance of the duodenal balloon occlusion method. PETCO_2_ was recorded every 10 min from the start of ESD to the end of ESD by nurses who were not notified about this study. CT scans, reconstructions of 3DCT images, and calculations of intestinal CO_2_ gas were performed respectively by separate radiology technicians who were not notified about this study.

All ESD procedures were performed by one JGES-certified endoscopist (H. M.) with experience of more than 150 cases annually. After all studies were finished, the data were analyzed by Drs. S. F. and H. K. This prospective clinical study was conducted after approval by the institutional ethics committee of Kagawa University Hospital and was enrolled with the University Hospital Medical Information Network (ID: 000003742).

Patients’ baseline statistics were analyzed using the unpaired *t*-test, Fisher’s exact test, and the Mann–Whitney U-test. Statistical significance was accepted for P values of <0.05. PETCO_2_ was analyzed every 10 min by repeated measure ANOVA (Graph Pad Prism 5).

## Results

### Study population

**Figure 2:**
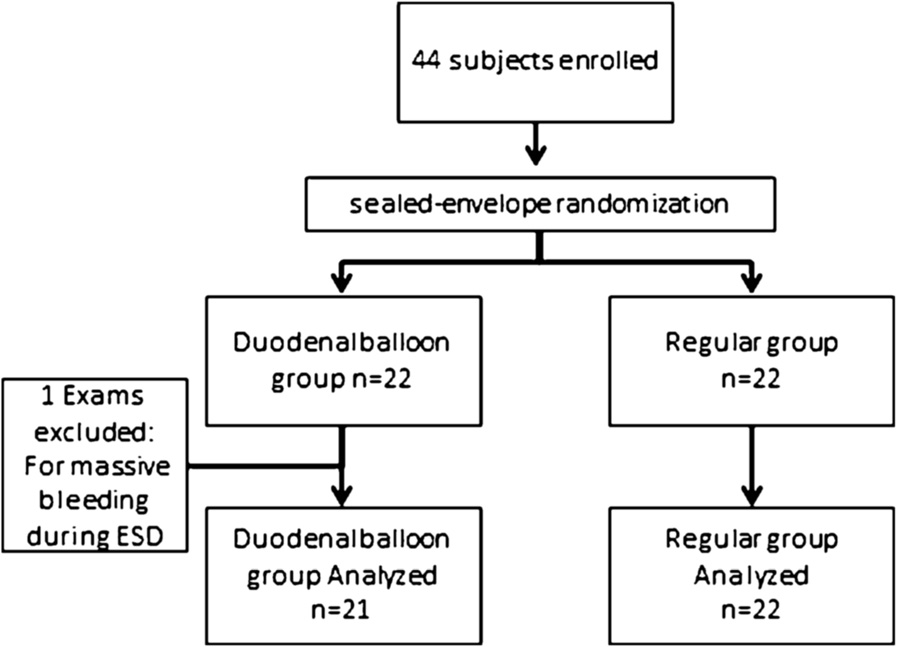
**A total of 44 subjects were enrolled. One subject did not continue treatment because of massive bleeding during ESD.** The remaining 43 subjects underwent subsequent endoscopic treatments according to the study protocol. Twenty-one subjects were randomly assigned to the duodenal balloon group and 22 to the placebo (regular) group.

A total of 44 subjects were enrolled in this study. One subject did not continue treatment because of massive bleeding during ESD. The remaining 43 subjects underwent subsequent endoscopic treatments according to the study protocol. Twenty-one subjects were randomly assigned to the duodenal balloon group and 22 to the placebo (regular) group. The allocation of the patients to the study groups and reasons for exclusion are shown in Figure 2. The baseline characteristics of the two groups were well matched, and no significant differences were found between the 43 patients with respect to age, gender, location of lesion, size of resected lesion, or procedure time (Table 1).

### Primary outcome

**Figure 3:**
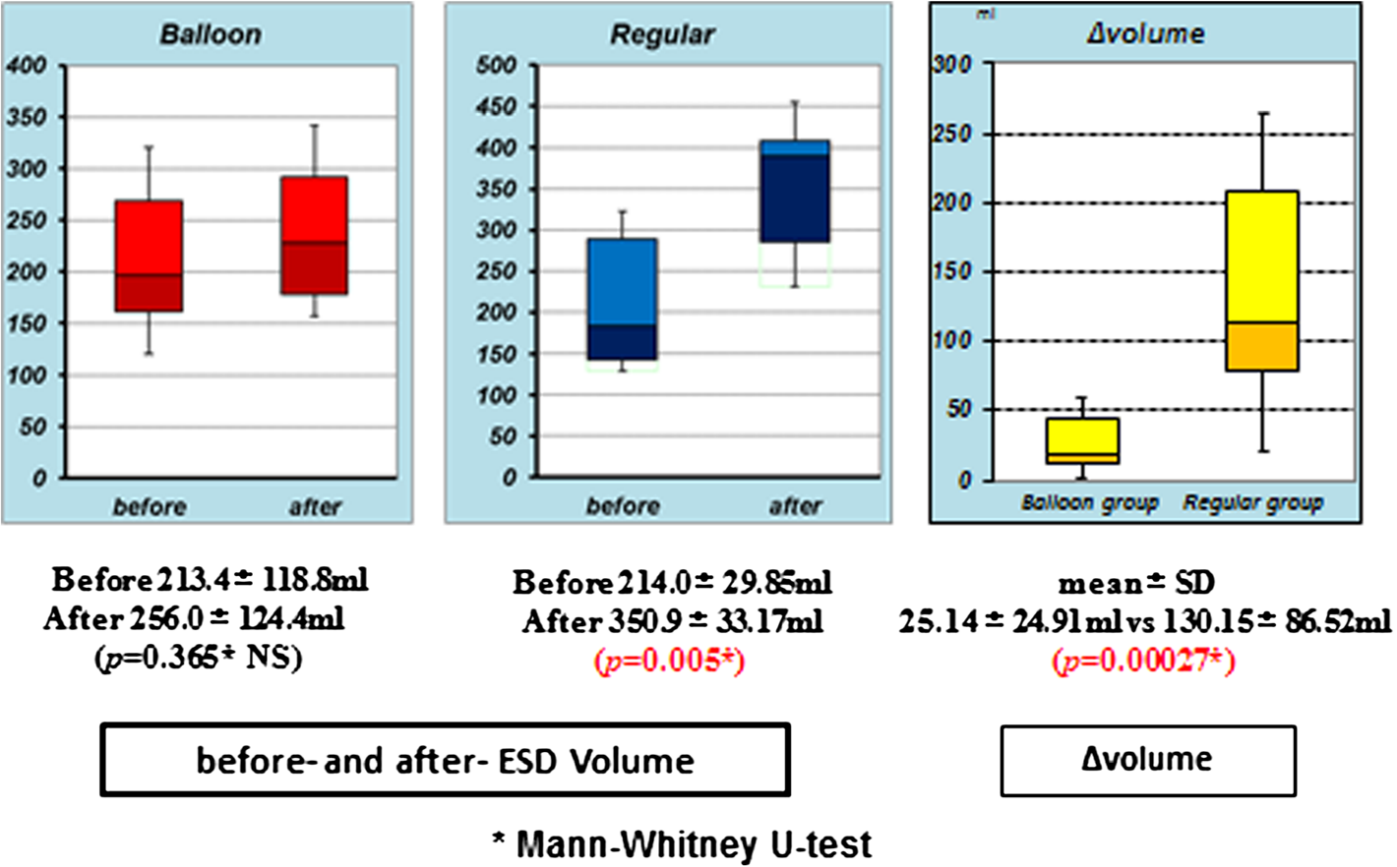
**The duodenal balloon group showed no significant difference in intestinal volume before (213.4 ± 118.8 mL) or after (256.0 ± 124.4 mL) ESD.** The regular group showed a significant difference before (214.0 ± 29.85 mL) and after (350.9 ± 33.17 mL) ESD (*P* = 0.005). The Δvolume before and after ESD was significantly lower in the duodenal balloon group (25.14 ± 24.91 mL) than in the regular group (130.15 ± 86.52 mL) (*P* = 0.00027).

The duodenal balloon group showed no significant difference in intestinal volume before (213.4 ± 118.8 mL) or after (256.0 ± 124.4 mL) ESD. On the other hand, the regular group showed a significant difference before (214.0 ± 29.85 mL) and after (350.9 ± 33.17 mL) ESD (*P* = 0.005). The amount of increase in intestinal volume (Δvolume) before and after ESD was significantly lower in the duodenal balloon group (25.14 ± 24.91 mL) than in the regular group (130.15 ± 86.52 mL) (*P* = 0.00027) (Figure 3).

### Secondary outcomes

**Figure 4:**
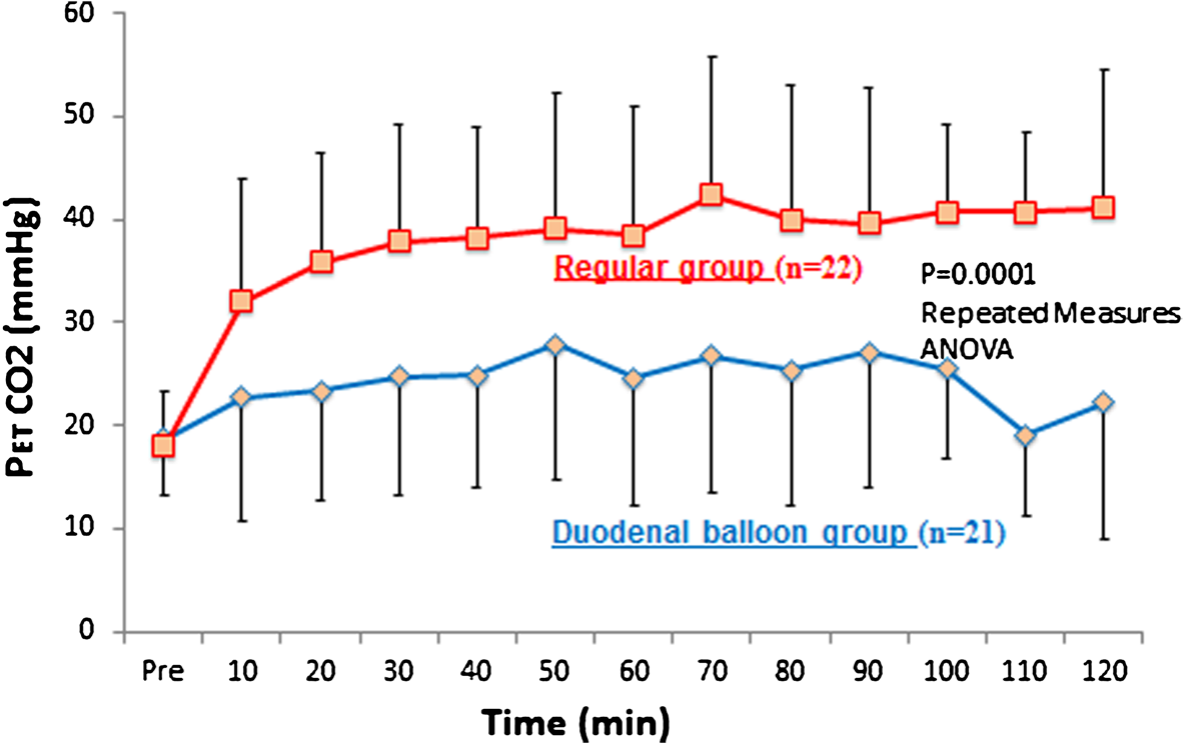
**The PETCO**_**2**_**level from 10 to 120 min after the start of ESD was significantly lower in the duodenal balloon group (22.1 ± 4.74 mmHg) than in the regular group (46.6 ± 1.69 mmHg) (*P*** **= 0.0001).**

The PETCO_2_ level from 10 to 120 min after the start of ESD was significantly lower in the duodenal balloon group (22.1 ± 4.74 mmHg) than in the regular group (46.6 ± 1.69 mmHg) (*P* = 0.0001) (Figure 4).

**Figure 5:**
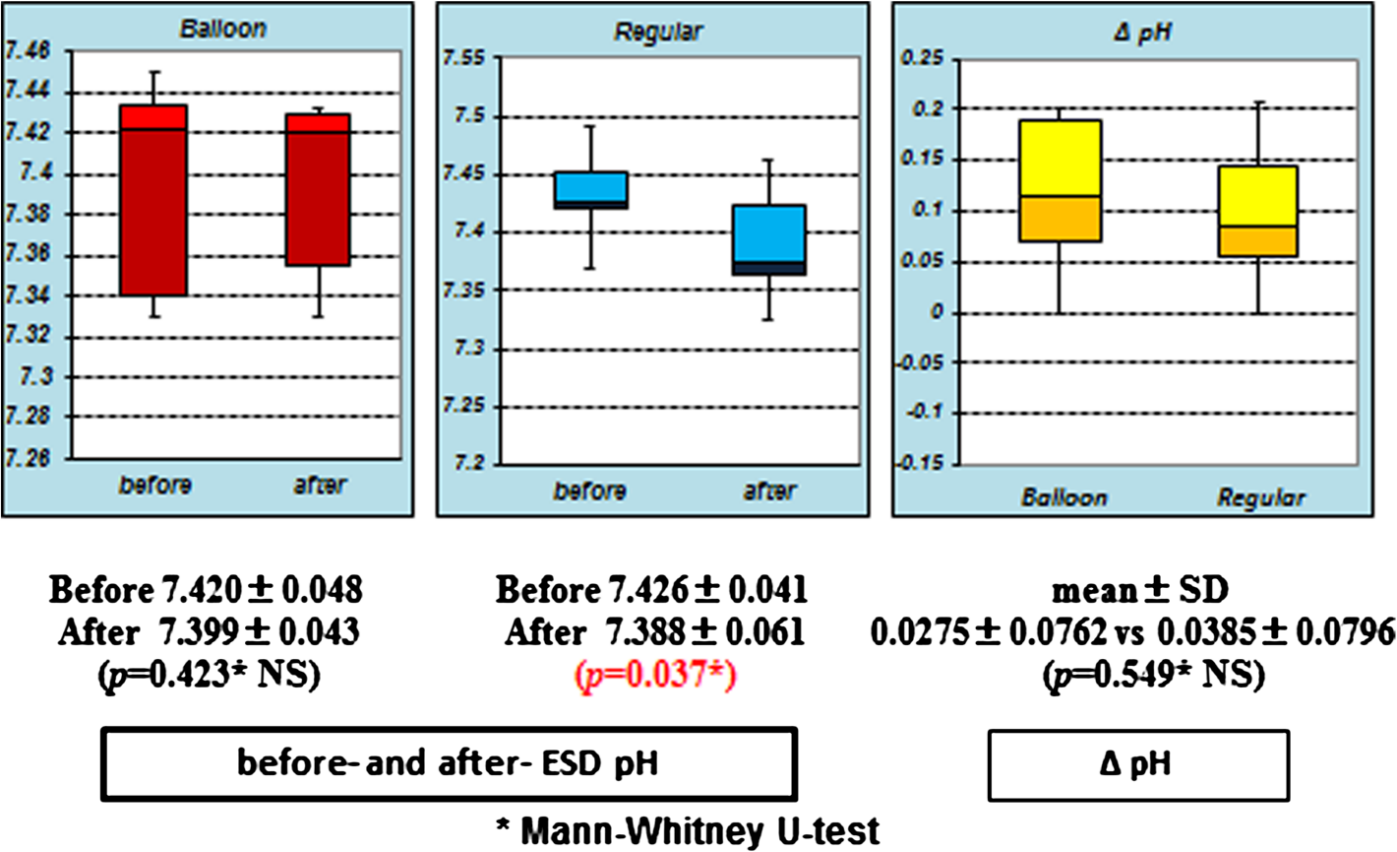
**The pH of the duodenal balloon group showed no significant difference (*P*** **= 0.423), but a significant difference was seen in the regular group (*P*** **= 0.037). The ΔpH showed no significant difference between the two groups (*P*** **= 0.549).**

In terms of arterial blood gas analysis before and just after ESD, pH of the duodenal balloon group showed no significant difference (*P* = 0.423), but a significant difference was seen in the regular group (*P* = 0.037). The ΔpH between the two groups showed no significant difference (*P* = 0.549) (Figure 5).

**Figure 6:**
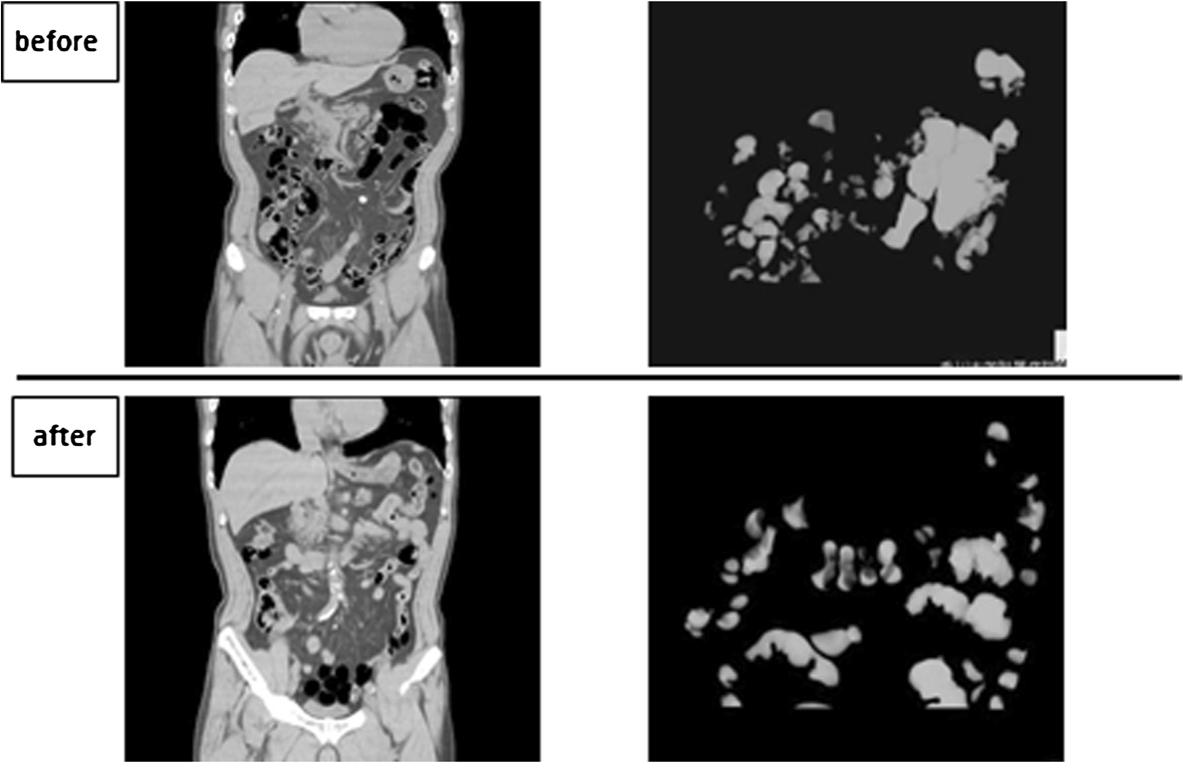
**Duodenal balloon group: CT coronal image & 3D image.** Two cases are depicted: Case 5 (duodenal balloon group) and Case 36 (regular group). 3DCT showed that the intestinal CO2 gas volume was relatively lower in the duodenal balloon group than in the regular group.

**Figure 7:**
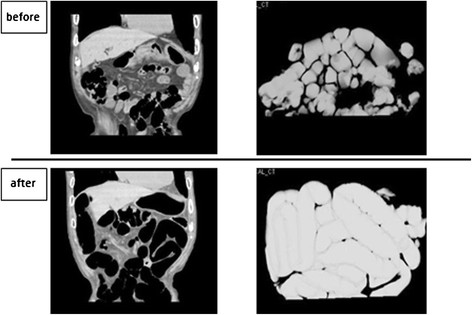
**Regular group: CT coronal image & 3D image.** Two cases are depicted: Case 5 (duodenal balloon group) and Case 36 (regular group). 3DCT showed that the intestinal CO2 gas volume was relatively lower in the duodenal balloon group than in the regular group.

As shown in Figures 6 and 7, which depict Case 5 (duodenal group) and Case 36 (regular group), the intestinal CO_2_ gas volume just before and after ESD was lower in the duodenal balloon group than in the regular group.

**Figure 8:**
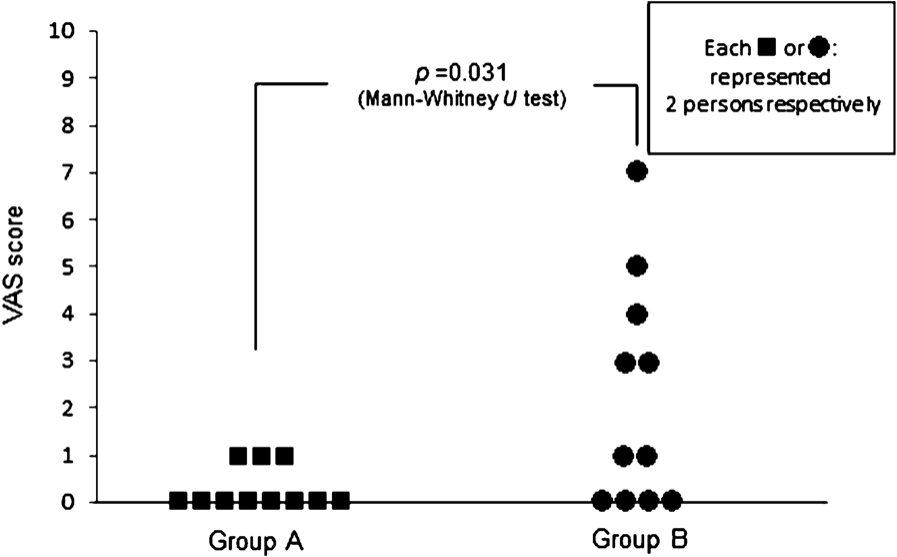
**The VAS scores (0 to 10) for the occurrence of nausea due to abdominal distension after ESD ranged from 0 to 1 in the duodenal balloon group and from 3 to 7 in the regular group, showing a significant difference between the two groups (*P*** **= 0.031).**

The VAS score for the occurrence of nausea due to abdominal distension after ESD ranged from 0 to 1 in the duodenal balloon group and from 3 to 7 in the regular group, revealing a significant difference (P = 0.031) (Figure 8).

## Discussion

The advantage of CO_2_-insufflated ESD for early colorectal cancer has been previously reported [10]. Complication associated with CO2 insufflation such as CO2 narcosis and gas embolism wasn’t seen [11]. Several studies has been reported about the influences of CO_2_ insufflation on the human body during ESD of the upper gastrointestinal (GI) tract, and it is well known that carbon dioxide is absorbed faster in the body than air and also that it is rapidly excreted through respiration without any complications [12]. And it is also reported that insufflation of CO2 than air during esophageal ESD significantly reduced postprocedural mediastinal emphysema [13]. In CO_2_-insufflated ESD for early colorectal cancer, CO_2_ gas is insufflated retrogradely, Bauhin’s valve functions as a backflow-prevention system, and the CO_2_ is absorbed only through the colonic mucosa.

In esophageal or gastric CO_2_-insufflated ESD, CO_2_ gas insufflated into the upper GI tract is widely distributed to and absorbed rapidly through not only the esophagus and stomach, but also other parts of the GI tract, such as the intestine. This suggests two potential effects of esophageal or gastric CO_2_-insufflated ESD on the human body.

First, massive insufflated CO_2_ gas into the stomach, duodenum, and intestine during or after ESD may worsen patient symptoms such as nausea and abdominal distension to some extent. Its volume effects may also influence patients’ respiratory functions. Our study reveals that a greater amount of CO_2_ gas was insufflated and remained after ESD without the duodenal balloon, and it affected patients’ symptoms (such as nausea) to some extent. We believe that the total insufflated CO_2_ gas volume should be controlled as little as possible whether or not it may have an unfavorable effect on the human body. A small CO_2_ gas volume is economical in terms of medical expense because it can reduce the cost of purchasing CO_2_.

Second, massive insufflated CO_2_ gas may affect patients’ blood gas analysis. In this study, changes in the CO_2_ level during ESD were examined by PETCO_2_ as measured by a capnometer that indirectly suggested the arterial blood CO_2_ concentration [23]. In the duodenal balloon group, the mean PETCO_2_ measured during ESD (22.1 ± 4.74 mmHg) was significantly lower than that of the regular group (46.6 ± 1.69 mmHg) (*P* = 0.0001). We did not observe significant differences in ΔpH between the two groups, but just after ESD in the regular group, there was a significant difference in pH (*P* = 0.037). This also implies that the arterial blood CO_2_ concentration levels in the regular group during ESD were higher than those of the balloon group. We sometimes perform ESD under general anesthesia if it will take longer because of the presence of a complicated lesion. When we performed ESD for a prolonged period under general anesthesia, some patients who underwent ESD with CO_2_ insufflation without the balloon showed dangerously high CO_2_ concentration levels (around 60 mmHg) during ESD. We were also advised that maintaining a high PETCO_2_ level during ESD should be avoided to prevent the risk of respiratory depression in patients with chronic obstructive respiratory disease (COPD). Therefore, in such COPD cases, the duodenal balloon method is preferable because it can maintain PETCO_2_ at low levels during ESD.

Furthermore, during procedures such as laparoscopic endoscopic cooperative surgery or hybrid natural orifice transluminal endoscopic surgery [24], a clear operational view without intestinal distension is helpful in obtaining a good laparoscopic field of view in our hospital.

## Conclusions

CO_2_-insufflated ESD for early esophageal or gastric cancer was proven safe for early colorectal cancer. However, the use of the duodenal balloon occlusion method in ESD for early esophageal or gastric cancer may reduce unnecessary insufflation of CO_2_ and prevent CO_2_ gas from flowing into the intestine, which can maintain a lower CO_2_ level in patients with COPD.

Limitations of this study were its single-center nature and small sample size.

## Competing interests

The authors declare that they have no competing interests.

## Authors’ contributions

Analysis and interpretation of the data was performed by HK, SF, NN, KR, MO and KI. Critical revision of the article for important intellectual content was performed by YS and TM. Final approval of the article was performed by TM. All authors read and approved the final manuscript.

## Pre-publication history

The pre-publication history for this paper can be accessed here:

http://www.biomedcentral.com/1471-230X/12/37/prepub
